# Tissue Adhesion-Anisotropic Polyrotaxane Hydrogels Bilayered with Collagen

**DOI:** 10.3390/gels7040168

**Published:** 2021-10-13

**Authors:** Masahiro Hakariya, Yoshinori Arisaka, Hiroki Masuda, Tetsuya Yoda, Atsushi Tamura, Takanori Iwata, Nobuhiko Yui

**Affiliations:** 1 Department of Periodontology, Graduate School of Medical and Dental Sciences, Tokyo Medical and Dental University (TMDU), Tokyo 113-8549, Japan; hakariya.peri@tmd.ac.jp (M.H.); iwata.peri@tmd.ac.jp (T.I.); 2 Department of Organic Biomaterials, Institute of Biomaterials and Bioengineering, Tokyo Medical and Dental University (TMDU), Tokyo 101-0062, Japan; arisaka.org@tmd.ac.jp (Y.A.); tamura.org@tmd.ac.jp (A.T.); 3 Department of Maxillofacial Surgery, Graduate School of Medical and Dental Sciences, Tokyo Medical and Dental University (TMDU), Tokyo 113-8549, Japan; masumfs@tmd.ac.jp (H.M.); yoda.mfs@tmd.ac.jp (T.Y.)

**Keywords:** polyrotaxane, hydrogel, tissue adhesive, collagen

## Abstract

Hydrogels are promising materials in tissue engineering scaffolds for healing and regenerating damaged biological tissues. Previously, we developed supramolecular hydrogels using polyrotaxane (PRX), consisting of multiple cyclic molecules threaded by an axis polymer for modulating cellular responses. However, since hydrogels generally have a large amount of water, their adhesion to tissues is extremely weak. Herein, we designed a bilayered hydrogel with a PRX layer and a collagen layer (PRX/collagen hydrogel) to achieve rapid and strong adhesion to the target tissue. The PRX/collagen hydrogel was fabricated by polymerizing PRX crosslinkers in water with placement of a collagen sponge. The differences in components between the PRX and collagen layers were analyzed using Fourier transform infrared spectroscopy (FT-IR). After confirming that the fibroblasts adhered to both layers of the PRX/collagen hydrogels, the hydrogels were implanted subcutaneously in mice. The PRX hydrogel without collagen moved out of its placement site 24 h after implantation, whereas the bilayer hydrogel was perfectly adherent at the site. Together, these findings indicate that the bilayer structure generated using PRX and collagen may be a rational design for performing anisotropic adhesion.

## 1. Introduction

Polyrotaxanes (PRXs) are supramolecular systems consisting of multiple cyclic molecules, an axis polymer, and end-capping molecules. The axis polymer threads into the cavities of cyclic molecules, and both ends of the polymer are capped with bulky molecules to prevent the de-threading of cyclic molecules [1]. A typical combination of cyclic molecules and axis polymers is α-cyclodextrin (α-CD) and poly (ethylene glycol) (PEG). Since α-CDs are non-covalently linked to the PEG axis, they can move freely along the polymer chain [2,3,4]. This molecular mobility has been utilized to design functional materials, such as stretcha ble hydrogels [5,6], self-healing materials [7], tissue engineering scaffolds [8,9], and drug delivery carriers [10]. We previously reported that PRX-coated surfaces with tunable molecular mobility provide a cellular environment for manipulating various cellular responses [8,11,12]. Recently, we fabricated a PRX hydrogel to incorporate molecular mobility into three-dimensional materials [13]. In particular, a PRX hydrogel cross-linked with methylated polyrotaxane end-capped with 4-vinylbenzyl groups (MePRX-VBn) was prepared. The cross-linking at the end of PRX may contribute to maintaining the molecular mobility of α-CDs even after hydrogel formation. The PRX hydrogel has cellular adhesive properties and may be used as a three-dimensional scaffold for manipulating cellular responses through the molecular mobility. However, synthetic hydrogels commonly contain a large amount of water, and even if they have cellular adhesion properties, their adhesion to biological tissues is significantly low, which limits their application in in vivo systems [14,15].

Natural polymers, such as fibrin and collagen, have high adhesiveness to cells and tissues [16,17,18]. Collagen, in particular, is a major component of the extracellular matrix and can be processed into various forms, such as sponges, gels, and films, which makes it one of the most suitable biomaterials for a wide range of applications [19]. For instance, a collagen-based sponge can absorb a large amount of tissue exudate, smoothly adhere to a moist wound bed, and maintain a moist environment to prevent mechanical trauma and bacterial infection. It is routinely used in dressing the wounds from severe burns and bedsores [19,20,21]. Collagen sponges, with their high adhesive properties, have also been reported for various applications as tissue engineering scaffolds [22,23]. However, collagen sponges present a challenge in the regeneration and repairing of complex tissues composed of multiple cell types. For instance, when regenerating periodontal tissue, which is a characteristic complex tissue composed of gingiva, periodontal ligament, alveolar bone, and cementum [24,25], it is necessary to inhibit the proliferation of gingival epithelium while promoting the proliferation of osteoblasts [26,27]. If the proliferation of gingival epithelial cells is enhanced using collagen sponges, the regeneration of alveolar bone would be inhibited. Therefore, to regenerate complex tissues, it is necessary not only to enhance cellular adhesion and proliferation but also to modulate various cellular responses, including proliferation, differentiation and gene expression.

To address these challenges, we designed a polyrotaxane hydrogel bilayered with a collagen sponge. The main feature of the bilayer hydrogel is that the properties of each layer can be separated. Hence, bifunctional hydrogels can be created without offsetting the individual abilities of collagen to rapidly and strongly adhere to biological tissues and polyrotaxane to modulate cellular responses. In the present study, bilayered hydrogels were prepared by polymerizing MePRX-VBn in water with the placement of a collagen sponge. The structure of the bilayered hydrogels was evaluated via scanning electron microscopy (SEM) and Fourier transform infrared (FT-IR) spectroscopy. To investigate cytocompatibility, mouse fibroblasts were cultured on hydrogels. The hydrogels were then implanted subcutaneously on the mice dorsa, and their adhesiveness was evaluated.

## 2. Results and Discussion

### 2.1. Characterization of Methylated Polyrotaxanes Capped with 4-vinylbenzyl Groups (MePRX-VBn)

**Figure 1:**
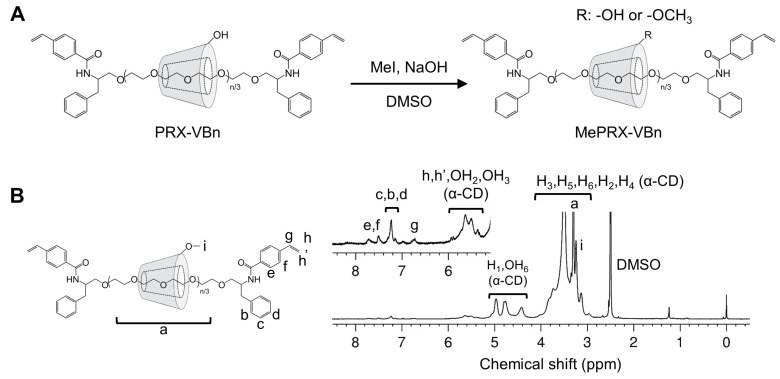
(**A**) Synthesis of methylated polyrotaxane with 4-vinylbenzyl groups (MePRX-VBn). (**B**) ^1^H nuclear magnetic resonance (NMR) spectra of Me_322_PRX-VBn in dimethyl sulfoxide (DMSO)-*d*_6_.

Unmodified PRX (the number of threading α-CDs = 22.3, *M*_n,PEG_ = 5000) precipitates in water due to intermolecular hydrogen bonding of the threading α-CD. Modification of the threaded α-CDs with methyl groups weakens their bonds, resulting in the water solubilization of PRX [28,29]. In the present study, MePRX-VBns with different degrees of methylation were prepared (Figure 1A). In the ^1^H nuclear magnetic resonance (NMR) spectrum in dimethyl sulfoxide (DMSO) of MePRX-VBn (Figure 1B), the characteristic proton peaks were observed at δ = 3.03–4.09 ppm (m, PEG backbone; H_2_, H_3_, H_4_, H_5_, and H_6_ of α-CD; and -O-CH_3_ of methyl groups), δ = 4.42 ppm (m, OH_6_, α-CD), δ = 4.66–5.12 ppm (m, H_1_ of α-CD), δ = 5.31–5.97 ppm (m, OH_3_ and OH_2_ of α-CD; and CH_2_=CH- of the vinyl group), δ = 6.76 ppm (m, CH_2_=CH- of the vinyl group), δ = 7.07–7.37 ppm (m, aromatics derived from 2-amino-3-phenylpropyl group), and δ = 7.41–7.84 ppm (m, aromatics derived from 4-vinylbenzyl group). After methylation, the peaks of methyl groups appeared, and the peaks of OH groups faded away. The number of methyl groups on MePRX-VBns was approximately 30.0, 112.3, and 322.0 (Me_30_PRX-VBn, Me_112_PRX-VBn and Me_322_PRX-VBn, respectively), as determined by the integration of the peaks at δ = 3.03–4.09 ppm and δ = 4.66–5.12 ppm derived from PEG and methylated α-CD from ^1^H NMR spectrum in DMSO before and after methylation (Table 1) (Figure S1 in Supporting Information).

**Figure 2:**
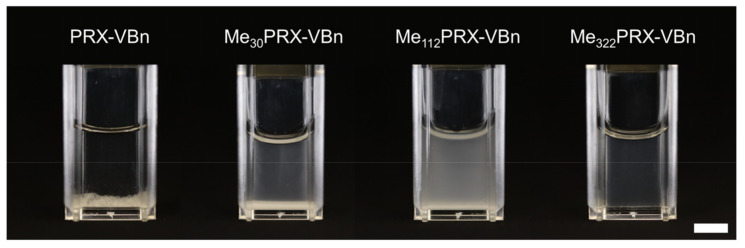
Photograph of polyrotaxane with 4-vinylbenzyl groups (PRX-VBn) and methylated PRX-VBn (MePRX-VBn) in water at 10 mg/mL (scale bar: 5 mm). MePRX-VBns with different degrees of methylation are abbreviated as Me*_X_*PRX-VBn, where *X* depicts the number of methyl groups.

To investigate water solubility, a series of PRX-VBns and MePRX-VBns were added at a concentration of 10 mg/mL in water (Figure 2). PRX-VBn and Me_30_PRX-VBn precipitated in water. Although no precipitation occurred in the Me_112_PRX-VBn solution, turbidity was observed. On the contrary, the Me_322_PRX-VBn solution was clear, which suggests that it was completely dissolved in water. Heterogeneous dispersion of monomers and crosslinkers can reduce the mechanical strength of hydrogels [30,31]. Therefore, in the present study, PRX hydrogels were fabricated using Me_322_PRX-VBn.

### 2.2. Fabrication and Characterization of PRX Hydrogels and PRX/Collagen Hydrogels

**Figure 3:**
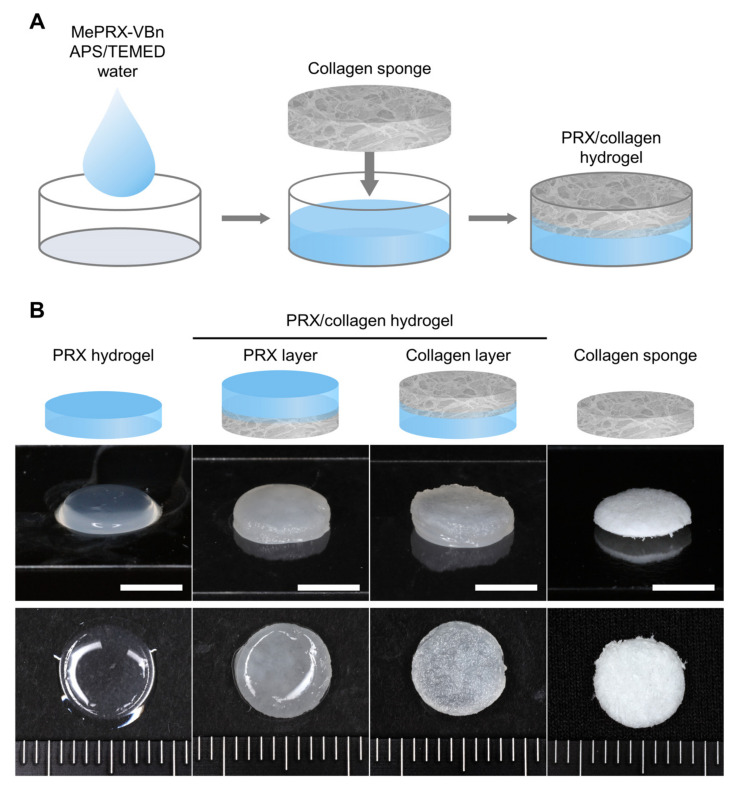
(**A**) Schematic of the fabrication procedure for hydrogels. (**B**) Photographs of polyrotaxane (PRX) hydrogels, PRX hydrogel side (PRX layer), and collagen sponge side (collagen layer) of PRX/collagen hydrogels and collagen sponges (scale bar: 5 mm).

PRX/collagen hydrogels were fabricated with Me_322_PRX-VBn and collagen sponges (Figure 3A). PRX hydrogels with Me_322_PRX-VBn were used as the control. In the PRX/collagen hydrogel, the surface of the PRX side (PRX layer) was smooth, while that of the collagen side (collagen layer) was rough (Figure 3B). These varying profiles are consistent with the surface profiles of PRX hydrogels and collagen sponges as single-component hydrogels. 

**Figure 4:**
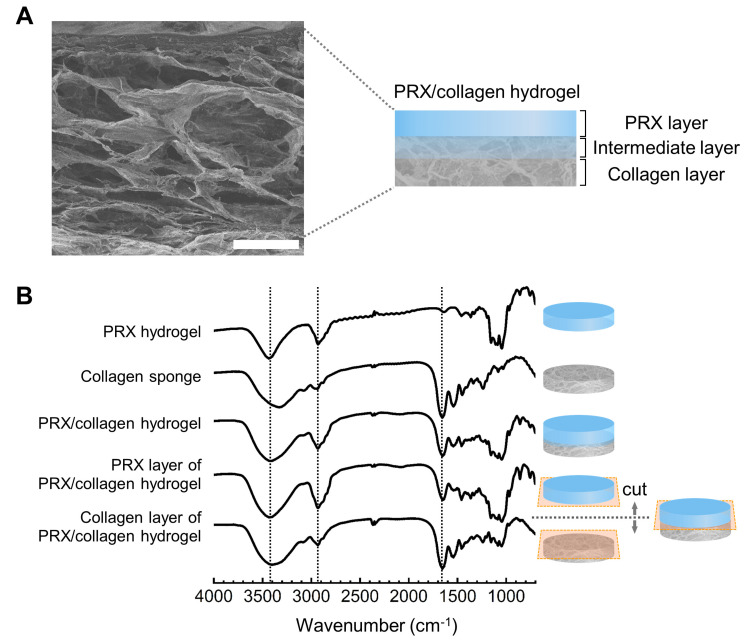
(**A**) SEM images of polyrotaxane (PRX)/collagen hydrogels (scale bar: 200 μm). (**B**) FT-IR spectra of PRX hydrogels, PRX/collagen hydrogels, the PRX layer in PRX/collagen hydrogels, the collagen layer in PRX/collagen hydrogels, and collagen sponges.

The microstructure of the PRX/collagen hydrogel was observed via SEM (Figure 4A). In the cross-section of the lyophilized PRX/collagen hydrogel, there was no boundary between the upper PRX layer and the lower collagen layer, suggesting that the network of each layer was interpenetrating in the intermediate layer. When the chemical composition of the lyophilized PRX/collagen hydrogel was measured via FT-IR (Figure 4B), peaks observed at 2930 cm^−1^ and 1655 cm^−1^ were attributed to the C-H stretching vibration of α-CD and PEG and the C=O stretching vibration of the peptide bond of collagen, respectively. The broad peaks at 3200 to 3700 cm^−1^ were attributed to the O-H stretching vibration of α-CD and collagen, and were observed in the spectra of both collagen and PRX layers. These suggest that the hydrogel is composed of PRX and collagen. To confirm the bilayered structure of PRX/collagen hydrogels, the PRX and collagen layers were separated from the lyophilized PRX/collagen hydrogels, followed by FT-IR analysis of each layer. In the FT-IR spectrum of the PRX layer, a strong peak at 2930 cm^−1^ was derived from PRX. This peak is hardly found in the spectrum of collagen sponge or collagen layer. On the other hand, in the FT-IR spectrum of the collagen layer, a distinctive peak at 1655 cm^−1^, derived from collagen, was observed. However, the spectra also indicated that each layer contained components from the other layer, suggesting that PRX and collagen formed an interpenetrated structure that was not completely separated. This result was supported by the results shown in photographs and SEM images.

### 2.3. Subcutaneous Implantation of the PRX/Collagen Hydrogel

**Figure 5:**
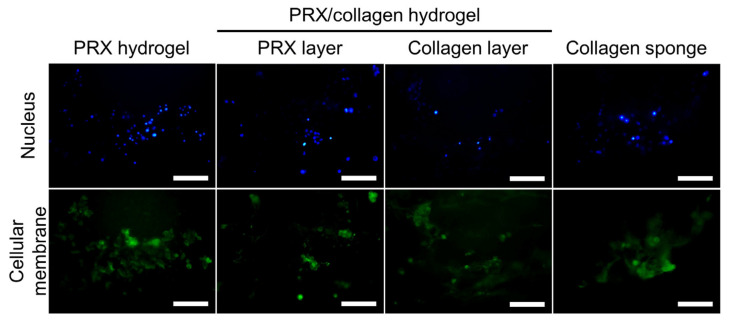
Fluorescent images of fibroblasts adhered to polyrotaxane (PRX) hydrogels, PRX/collagen hydrogels (the PRX and collagen layers), and collagen sponges. Scale bar: 100 μm. Blue: nucleus, green: cellular membranes.

Prior to in vivo implantation of PRX/collagen hydrogels, cellular adhesion on the PRX and collagen layers was analyzed. When mouse fibroblasts were cultured on the PRX and collagen layers of the PRX/collagen hydrogel, PRX hydrogel, and collagen sponge for 3 days, cellular adhesion was observed on all the hydrogels (Figure 5). The methyl group modified on α-CD promotes the adsorption of serum-derived cellular adhesion proteins [32,33], which may contribute to the promotion of cellular adhesion of PRX/collagen hydrogels. These results suggest that the PRX/collagen hydrogel is a promising implantable scaffold, with cytocompatibility.

**Figure 6:**
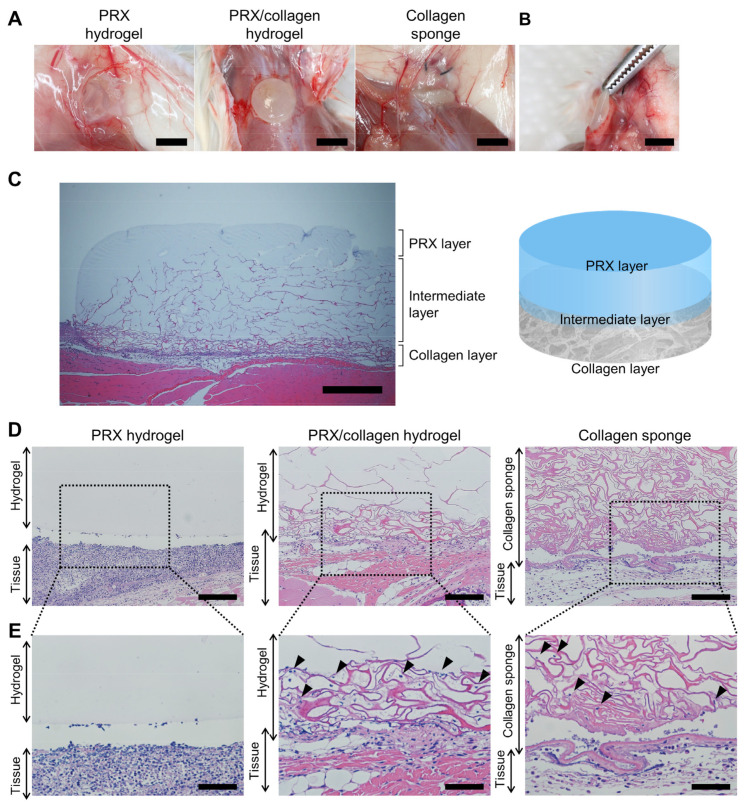
(**A**) Photographs of the polyrotaxane (PRX) hydrogel, PRX/collagen hydrogel, and collagen sponge after subcutaneous implantation on mice for 24 h (scale bar: 5 mm). (**B**) Photograph of PRX/collagen hydrogel tightly adhering to the muscle (scale bar: 5 mm). (**C**) Hematoxylin and eosin (HE) staining of a cross-section of PRX/collagen hydrogel (scale bar: 0.5 mm). (**D**) Histological observation of PRX hydrogels, PRX/collagen hydrogels, and collagen sponges and the surrounding tissues after subcutaneous implantation for 24 h; scale bar: 100 μm, (**E**) scale bar: 50 μm. The black arrows indicate migrated cells in the collagen layer.

To assess tissue adhesion, PRX/collagen hydrogels were placed on the dorsal muscle tissues of mice. After 24 h, the mice were sacrificed, and the dorsal pocket was re-opened (Figure 6A). PRX hydrogels and collagen sponges were used as controls. The PRX hydrogels moved away significantly from the implantation position and adhered to the skin tissue side. All the collagen sponges adhered to both the skin and muscle sides. Collagen strongly adhered to the tissue in a short time and did not move from the implanted position. The PRX/collagen hydrogel was placed on the muscle tissue, with the collagen side down. All the PRX/collagen hydrogels adhered to the muscle side, and no movement from the implanted position was observed even when they were pulled with tweezers (Figure 6B). This suggests that these hydrogels rapidly adhered to tissues through the collagen layer. The PRX layer of the PRX/collagen hydrogel also adhered to the skin side, but the adhesion was extremely weak, and the hydrogel was easily peeled off. Although cell-adhesive PRX hydrogels have the potential to adhere to tissues, a quicker and stronger cell-adhesive ability is required for implantation into a specific position of the target tissue. All the hydrogels and surrounding tissues were collected by incision and stained with hematoxylin and eosin (HE) for histological observation. Interestingly, since the collagen sponge was also HE-stained, the HE-stained cross-sectional images revealed the detailed structure of the PRX/collagen hydrogel (Figure 6C). The top surface of the PRX/collagen hydrogel consisted only of PRX hydrogel, the bottom surface consisted only of collagen sponge, and the intermediate layer between PRX and collagen layers was an interpenetrating network of the PRX hydrogel and collagen sponge. Although the gel may have shrunk due to HE staining, the thickness of the PRX layer, the interlayer between the PRX layer and the collagen layer, and the collagen layer were approximately 0.3, 0.7, and 0.1 mm, respectively. These structures are consistent with the SEM images and FT-IR analysis results. To reveal the detailed adhesion conditions of PRX/collagen hydrogels, histological observations were conducted, as shown in Figure 6D,E. The PRX hydrogels detached from the tissue, since none of the cells infiltrated the PRX hydrogel. The surface of the collagen sponge was tightly adhered with the tissue. The surrounding cells migrated into the collagen sponge, and tissue exudate deposition was observed. In the PRX/collagen hydrogel, the surface of the collagen layer tightly adhered with the tissue, and cellular migration and tissue exudate deposition were observed in the collagen layer. Hence, collagen sponges and the PRX/collagen induced migration of cells into the collagen layer and deposited exudates from the tissue, thereby resulting in strong adhesion to the host tissue.

## 3. Conclusions

An implantable bilayered hydrogel containing a PRX hydrogel layer and a collagen sponge layer was successfully synthesized and confirmed anisotropic adhesion to the target tissue via collagen layers. In the future, the molecular mobility of implantable PRX hydrogels may realize in vivo control of cellular functions, including cell proliferation and differentiation. Moreover, bilayered hydrogels of polyrotaxane and collagen will be promising biomaterials as an anisotropic tissue engineering scaffold with tissue adhesion, to repair composite tissues such as periodontal tissues. The structural concept of bilayer hydrogels is effective for designing biomaterials because it allows the coexistence of two different functions in one material. This method could be beneficial for successfully utilizing PRX hydrogels for biomedical applications.

## 4. Materials and Methods

### 4.1. Materials

PRX-VBn consisting of α-CDs and PEG (*M*_n_ = 5000) capped with 4-vinylbenzyl groups was synthesized as previously described by Arisaka et al. [13] (Figures S2 and S3 in Supporting Information). Iodomethane (MeI) was purchased from Tokyo Chemical Industry (Tokyo, Japan). Methanol (MeOH) was purchased from Kanto Chemical (Tokyo, Japan). Collagen sponges for 35 mm culture dishes were purchased from Koken (Tokyo, Japan). Fetal bovine serum (FBS) was purchased from Gibco BRL, Life Technologies (Carlsbad, CA, USA). Ammonium persulfate (APS), butorphanol tartrate, dimethyl sulfoxide (DMSO), Dulbecco’s modified Eagle’s medium (high glucose, with L-glutamine, phenol red, and sodium pyruvate) (D-MEM), 100 U/mL penicillin, 100 µg/mL streptomycin, medetomidine hydrochloride, midazolam, phosphate buffered saline (PBS), sodium hydroxide (NaOH) and *N*,*N*,*N’*,*N’*-tetramethylene diamine (TEMED) were purchased from FUJIFILM Wako Pure Chemical Corporation (Osaka, Japan). The BALB/3T3 clone A31, a fibroblast cell line established from mouse embryos, was obtained from the American Type Culture Collection (Manassas, VA, USA). BALB/c mice were obtained from Sankyo Labo Service (Tokyo, Japan).

### 4.2. Synthesis of Methylated PRX-VBn

PRX-VBns were prepared according to our previously described method [34], with a minor modification (Figure S2 in Supporting Information). The typical procedure for synthesis of methylated PRX-VBns (Me_322_PRX-VBn in Table 1) is as follows: briefly, PRX-VBn (1.0 g, 36 µmol) was dissolved in dehydrated DMSO (81 mL). NaOH (1.8 g, 44 mmol) and MeI (0.90 mL, 15 mmol) were added to the solution. After stirring for 1 h at 25 °C, the reaction solution was quenched with MeOH and purified by dialysis for 3 days (Spectra/Por 6, molecular weight cut-off of 3500) (Spectrum Laboratories, Rancho Dominguez, CA). The polymer was freeze-dried to yield MePRX-VBn as a powder (770 mg). MePRX-VBn with different degrees of methylation was synthesized by varying the feed MeI/OH_PRX_ ratios (0.11, 0.44, and 0.99).

### 4.3. Characterization of Polyrotaxane

^1^H NMR spectra in DMSO-*d*_6_ were recorded using a Bruker Avance III 400 MHz spectrometer (Bruker BioSpin, Rheinstetten, Germany). 

### 4.4. Fabrication of PRX Hydrogel and PRX/Collagen Hydrogel

To eliminate oxygen, the water for each solution was bubbled with nitrogen gas for 30 min. Me_322_PRX-VBn crosslinkers were dissolved in water in a 1.5 mL reaction tube. The concentration of the solution was 100 mg/mL. After mixing the Me_322_PRX-VBn solution, 10 µL of APS solution (10 mg/mL in water) was added to the reaction tube as an initiator. Separately, 20 µL of TEMED was dissolved in 1 mL water, and 10 µL of TEMED solution was added to the reaction tube as a catalyst. For the PRX/collagen hydrogel, a collagen sponge punched with a diameter of 7 mm was placed on the reaction solution. The solution was partially soaked in a sponge and polymerized at 24 °C in a nitrogen atmosphere for 24 h.

### 4.5. FT-IR Analysis

An FT-IR spectrometer (Spectrum 100, Perkin Elmer, Waltham, MA, USA) was used to examine the differences in the main functional groups on each layer of PRX/collagen hydrogels. PRX hydrogels, PRX layers of PRX/collagen hydrogels, collagen layers of PRX/collagen hydrogels, PRX/collagen hydrogels, and collagen sponges were examined. PRX layers and collagen layers of PRX/collagen hydrogels were prepared by dividing PRX/collagen hydrogels into upper and lower layers. For measurements, all the samples were ground with potassium bromide (KBr) (FUJIFILM Wako) and compressed to prepare pellets. All spectra were recorded in the frequency range of 700–4000 cm^−1^ at a resolution of 4 cm^−1^ and analyzed using the Spectrum software (Perkin Elmer).

### 4.6. SEM Analysis

To investigate the morphology of PRX/collagen hydrogels, dried hydrogels were observed using a Hitachi S-3400NX SEM (Hitachi, Japan). The dried hydrogels were sputter-coated with gold particles (SC-701AT, ELIONIX, Tokyo, Japan) and observed at an accelerating voltage of up to 15 kV.

### 4.7. Culturing of Fibroblasts on PRX Hydrogel and PRX/Collagen Hydrogel

The BALB/3T3 clone A31, a mouse fibroblast cell line established from embryos, was obtained from the American Type Culture Collection (Manassas, VA, USA). BALB/3T3 cells were cultured in Dulbecco’s modified Eagle’s medium (high glucose, with L-glutamine, phenol red, and sodium pyruvate) (FUJIFILM Wako), supplemented with 10% fetal bovine serum (Thermo Fisher Scientific, Waltham, MA, USA), 1% penicillin (100 U/mL) (FUJIFILM Wako), and streptomycin (100 μg/mL) (FUJIFILM Wako), in a humidified 5% CO_2_ atmosphere at 37 °C. To evaluate cellular adhesion on the hydrogels, BALB/3T3 cells were cultured on the hydrogels, and cellular adhesion was examined. Before seeding the cells, the hydrogels and collagen sponge were sterilized by ultraviolet irradiation for 20 min.

BALB/3T3 cells were seeded on the PRX hydrogels, the PRX and collagen layers of PRX/collagen hydrogels and the collagen sponge, at a density of 10 × 10^4^ cells per sample, and cultured in medium at 37 °C in a humidified atmosphere with 5% CO_2_ for 3 days. To stain the cellular membranes, adherent cells were incubated with the reagents of the Cell Navigator Cell Plasma Membrane Staining Kit (AAT Bioquest, Inc., Sunnyvale, CA, USA) (1:500). After washing the cells with PBS, nuclei were stained with Hoechst 33342 (Dojindo, Kumamoto, Japan) (1:500) and washed with PBS. The adherent cells were observed using a fluorescence microscope (IX71, Olympus, Tokyo, Japan) equipped with a digital camera (DP80, Olympus), and fluorescence images were captured using the CellSens standard software (Olympus).

### 4.8. In Vivo Subcutaneous Implantation of PRX Hydrogel and PRX/Collagen Hydrogel

Eight-week-old male BALB/c mice were obtained from Sankyo Labo Service (Tokyo, Japan). All animal experiments were approved by the Animal Care Committee of the Experimental Animal Center at Tokyo Medical and Dental University (approval number: A2021–029C2). Mice were anesthetized by subcutaneous administration of the three mixed solutions. The solution contained 0.75 mg/kg of medetomidine hydrochloride (FUJIFILM Wako), 4 mg/kg of midazolam (FUJIFILM Wako), and 5 mg/kg of butorphanol tartrate (FUJIFILM Wako) at a dose of 10 µL/g of body weight. Two dorsal skin incisions were performed, and the skin was separated from the muscles. Two subcutaneous pockets were created per animal. PRX hydrogels, PRX/collagen hydrogels or collagen sponges were implanted into the dorsal subcutaneous pockets, the incisions were sutured, and the animals were returned to their cages. After 24 h, all animals were anesthetized using the same procedures and sacrificed. Incisions were made on the skin to open the dorsal skin flap, and samples and surrounding tissues were collected for histological analysis.

For histological analysis, all samples were fixed in 4% paraformaldehyde (FUJIFILM Wako) and processed for hematoxylin and eosin (HE) staining at the Biopathology Institute (Oita, Japan). The histological sections were imaged using an IX-71 microscope (Olympus, Tokyo, Japan) equipped with a DP-80 dual CCD digital camera (Olympus).

## Figures and Tables

**Table 1:** Characterization of PRX-VBn and MePRX-VBns.

Code *^a^*	*M*_n_ of PEG Axle	Number of Threaded α-CDs on PRX	*M*_n,NMR_ of PRX	Number of Methyl Groups on PRX
PRX-VBn	5000	22.3	27,200	0
Me_30_PRX-VBn	5000	22.3	27,600	30.0
Me_112_PRX-VBn	5000	22.3	28,800	112.3
Me_322_PRX-VBn	5000	22.3	31,700	322.0

*^a^* PRX-VBn with methyl groups are abbreviated as Me*_X_*PRX-VBn, where *X* depicts the number of methyl groups. PEG, poly(ethylene glycol); α-CD, α-cyclodextrin; PRX, polyrotaxane; PRX-VBn, polyrotaxane end-capped with 4-vinylbenzyl groups.

## Data Availability

The data in this work are available from the corresponding author upon reasonable request.

## Supplementary Materials

The following are available online at https://www.mdpi.com/article/10.3390/gels7040168/s1, Figure S1. ^1^H nuclear magnetic resonance (NMR) spectra of Me_30_PRX-VBn (A) and Me_112_PRX-VBn (B) in dimethyl sulfoxide (DMSO)-*d*_6_, Figure S2. Synthesis of polyrotaxanes capped with 4-vinylbenzyl groups and subsequent modification of methyl groups, Figure S3. Size exclusion chromatography (SEC) chart for polyrotaxane capped with 4-vinylbenzyl groups (PRX-VBn) and methylated PRX-VBn with 322 methyl groups (Me_322_PRX-VBn). SEC was performed using Prominence-i LC-2030 Plus (Shimadzu, Kyoto, Japan) equipped with a RID-20A refractive index detector (Shimadzu) and a combination of TSK gel^®^ α-4000 and α-2500 columns (300 mm length, 7.8 mm internal diameter) (Tosoh, Tokyo, Japan). The system was eluted with DMSO containing 10 mM lithium bromide as an eluent, at a flow rate of 0.35 mL/min at 60 °C. Poly(ethylene glycol) standards were used for calibration.
