# Supplementary material for: Tissue Adhesion-Anisotropic Polyrotaxane Hydrogels Bilayered with Collagen
Source: Gels. 2021 Oct 13;7(4):168. doi: 10.3390/gels7040168 (PMC8544508; doi:10.3390/gels7040168)
Supplement: Supplementary file 1 [file gels-07-00168-s001.zip › gels-1419601-supplementary.pdf]

Supporting Information

Tissue Adhesion-Anisotropic Polyrotaxane Hydrogels Bi-layered with Collagen

Masahiro Hakariya, Yoshinori Arisaka, Hiroki Masuda, Tetsuya Yoda, Atsushi Tamura, Takanori Iwata and Nobuhiko Yui*

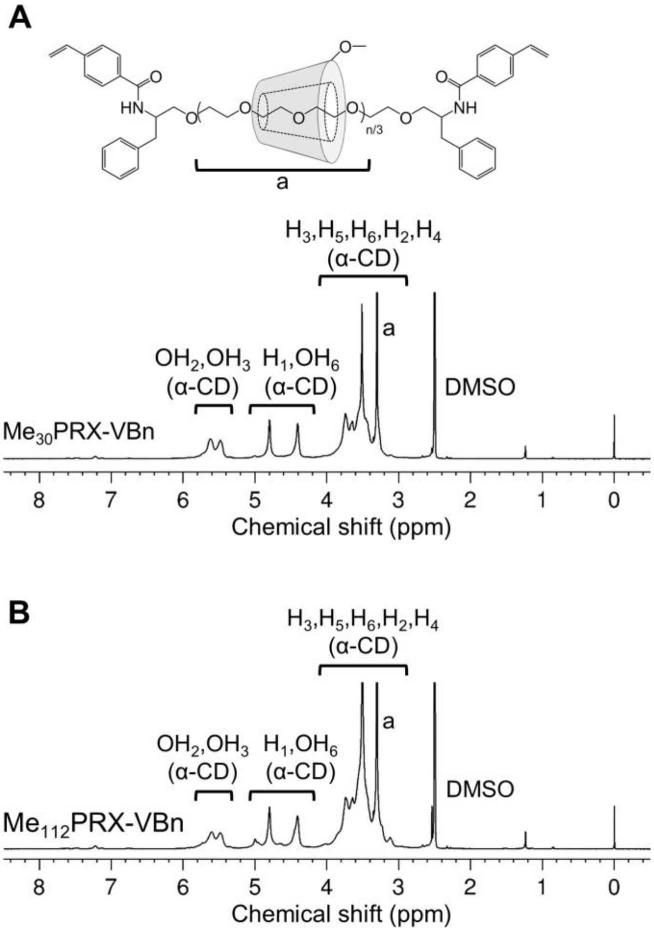

Figure S1. ^{1}H nuclear magnetic resonance (NMR) spectra of Me_{30}PRX-VBn (A) and Me_{112}PRX-VBn (B) in dimethyl sulfoxide (DMSO)-d_6.

Synthesis of Polyrotaxane end-capped with 4-vinylbenzyl groups

Materials

Hydroxy-terminated PEG (PEG-OH) (M_n = 5,000, M_w/M_n = 1.11) and 4-vinylbenzoic acid were purchased from Sigma-Aldrich (St. Louis, MO). Phenylalaninol and triethylamine (TEA) were purchased from Tokyo Chemical Industry (Tokyo, Japan). *N,N*-Dimethylformamide (DMF), dimethyl sulfoxide (DMSO), methanesulfonyl chloride (MsCl), methanol (MeOH), and tetrahydrofuran (THF) were purchased from Kanto Chemical (Tokyo, Japan). 4-(4,6-dimethoxy-1,3,5-triazin-2-yl)-4-methylmorpholinium chloride (DMT-MM) and sodium hydride (NaH) were purchased from FUJIFILM Wako Pure Chemical Corporation (Osaka, Japan). Diethyl ether was purchased from the Showa Ether Corporation (Tokyo, Japan).

Methods

Polyrotaxanes capped with 4-vinylbenzoic acid (PRX-VBn) were prepared according to our previously described method [1] (**Figure S2** in Supporting Information). Briefly, PEG-OH (20.0 g, 4.3 mmol), TEA (9.3 mL, 66.4 mmol), and MsCl (3.4 mL, 44.4 mmol) were allowed to react in anhydrous THF (115 mL) for 5 h at 23 °C. The solution was added dropwise to diethyl ether to precipitate the polymer. After drying, α,ω-bismesyl-PEG (PEG-Ms) was obtained as a powder (20.1 g, 97.3% yield). All the PEG-Ms (20.1 g, 4.1 mmol) were dissolved in anhydrous DMF (100 mL) under a nitrogen atmosphere. Separately, NaH (2.0 g, 83.4 mmol) and phenylalaninol (6.4 g, 42.3 mmol) were allowed to react in DMF (150 mL) for 30 min in a nitrogen atmosphere. Both solutions were stirred for 24 h at 23 °C under a nitrogen atmosphere. The reaction solution was added dropwise to diethyl ether to

precipitate the polymer. After filtration and drying, bis(2-amino-3-phenylpropyl) PEG (PEG-Phe-NH_2) was obtained as a powder (12.2 g, 59.4% yield). PEG-Phe-NH_2 (5.0 g, 1.0 mmol) was slowly added to a saturated aqueous solution of α-CD (25.0 g, 25.7 mmol) in water (172 mL), and the turbid solution was stirred for 24 h at 25 °C. After collection via centrifugation, the precipitate was freeze-dried for 24 h to obtain pseudo-polyrotaxane (pseudo-PRX-NH_2). For capping both terminals of the pseudo-polyrotaxane, a solution of 4-vinylbenzoic acid (3.0 g, 20.1 mmol) and DMT-MM (5.7 g, 20.1 mmol) in MeOH (220 mL) was slowly added to the pseudo-PRX-NH_2 and allowed to react at 25 °C for 24 h. The precipitate was collected via centrifugation, shaken with methanol, dissolved in dimethyl sulfoxide, and reprecipitated in water. The product was collected after centrifugation and freeze-dried to obtain polyrotaxane capped with 4-vinylbenzyl groups (PRX-VBn) as a powder (11.2 g, 46.3% yield; calculated based on the molecular equivalents of the PEG axle). Although α-CDs associate with PEG in water, the complexes disassociate in DMSO. The formation of polyrotaxane by capping the end of PEG with a 4-vinylbenzyl group was confirmed by SEC analysis using DMSO (Figure S3 in Supporting Information).

Reference

[1] Arisaka, Y.; Tonegawa, A.; Tamura, A.; Yui, N. Terminally cross-linking polyrotaxane hydrogels applicable for cellular mi-croenvironments. J. Appl. Polym. Sci. 2021, 138, 49706, doi: 10.1002/app.49706.

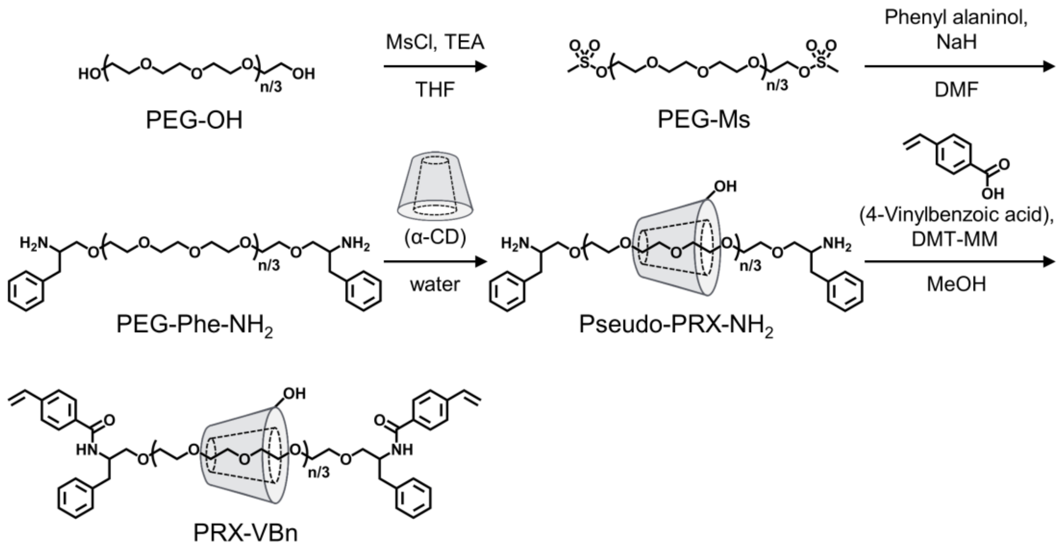

Figure S2. Synthesis of polyrotaxanes capped with 4-vinylbenzyl groups and subsequent modification of methyl groups

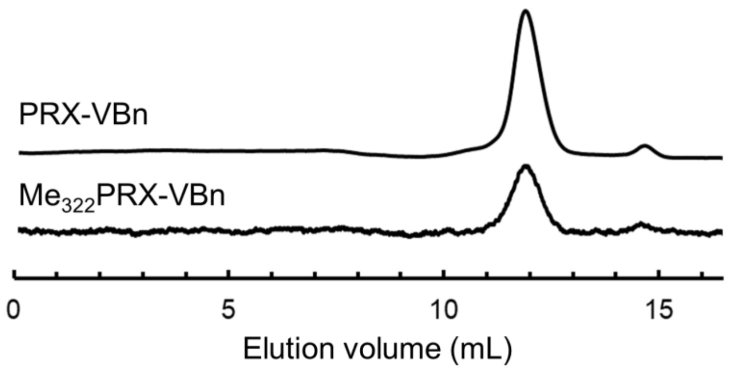

Figure S3. Size exclusion chromatography (SEC) chart for polyrotaxane capped with 4-vinylbenzyl groups (PRX-VBn) and methylated PRX-VBn with 322 methyl groups (Me_{322}PRX-VBn). SEC was performed using Prominence-i LC-2030 Plus (Shimadzu, Kyoto, Japan) equipped with a RID-20A refractive index detector (Shimadzu) and a combination of TSK gel® α-4,000 and α-2,500 columns (300 mm length, 7.8 mm internal diameter) (Tosoh, Tokyo, Japan). The system was eluted with dimethyl sulfoxide containing 10 mM lithium bromide as an eluent, at a flow rate of 0.35 mL/min at 60 °C. Poly(ethylene glycol) standards were used for calibration.
